# Supplementary material for: Hyperthermic Intravesical Chemotherapy (HIVEC) Using Epirubicin in an Optimized Setting in Patients with NMIBC Recurrence after Failed BCG Therapy
Source: Cancers (Basel). 2024 Apr 2;16(7):1398. doi: 10.3390/cancers16071398 (PMC11011040; doi:10.3390/cancers16071398)

Supplementary Figure S1. (A) CSS and (B) OS after device assisted HIVEC for NMIBC recurrence after failed BCG therapy using conductive heating of Epirubicin in an optimized setting.

A

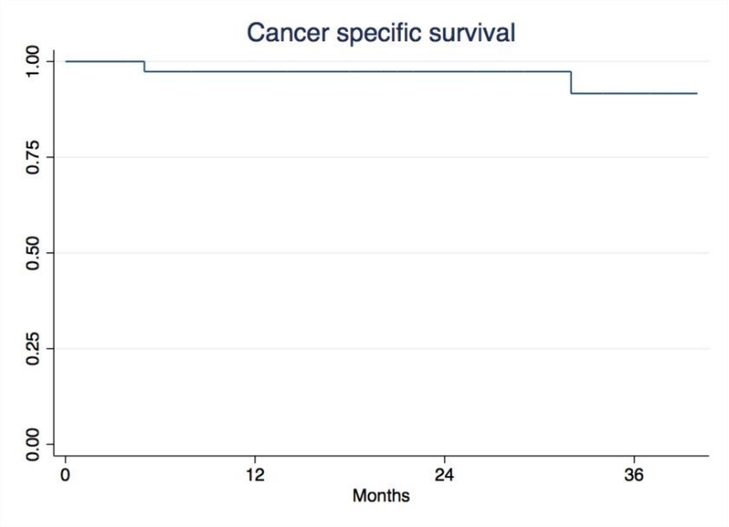

B

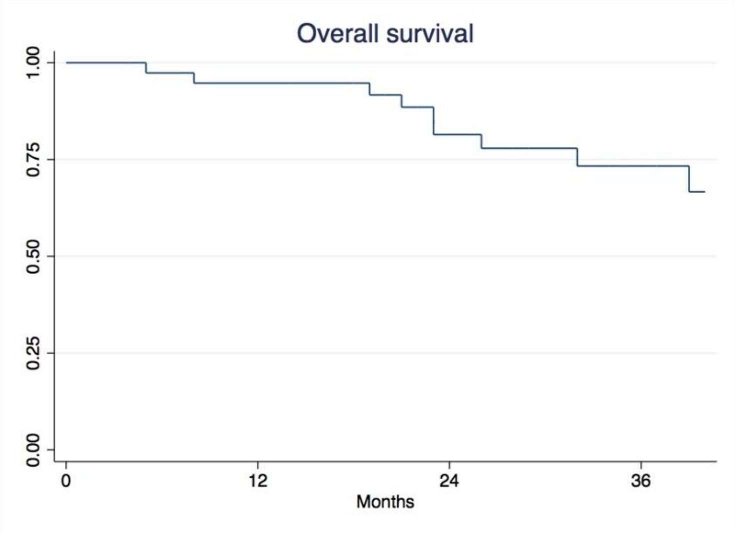

Supplement: Supplementary file 1 [file cancers-16-01398-s001.zip › cancers-2905801-supplementary.pdf]
